# Prediagnostic Serum Biomarkers as Early Detection Tools for Pancreatic Cancer in a Large Prospective Cohort Study

**DOI:** 10.1371/journal.pone.0094928

**Published:** 2014-04-18

**Authors:** Brian M. Nolen, Randall E. Brand, Denise Prosser, Liudmila Velikokhatnaya, Peter J. Allen, Herbert J. Zeh, William E. Grizzle, Aleksey Lomakin, Anna E. Lokshin

**Affiliations:** 1 University of Pittsburgh Cancer Institute, Pittsburgh, Pennsylvania, United States of America; 2 Department of Medicine, Division of Gastroenterology, Hepatology and Nutrition, University of Pittsburgh and University of Pittsburgh Medical Center, Pittsburgh, Pennsylvania, United States of America; 3 Department of Surgery, Memorial Sloan-Kettering Cancer Center, New York, New York, United States of America; 4 Department of Surgery, School of Medicine, University of Pittsburgh, Pittsburgh, Pennsylvania, United States of America; 5 Department of Pathology, University of Alabama at Birmingham, Birmingham, Alabama, United States of America; 6 Department of Physics, Massachusetts Institute of Technology, Cambridge, Massachusetts, United States of America; 7 Department of Medicine, School of Medicine, University of Pittsburgh, Pittsburgh, Pennsylvania, United States of America; 8 Department of Pathology, School of Medicine, University of Pittsburgh, Pittsburgh, Pennsylvania, United States of America; 9 Department of Ob/Gyn, School of Medicine, University of Pittsburgh, Pittsburgh, Pennsylvania, United States of America; Centro Nacional de Investigaciones Oncológicas (CNIO), Spain

## Abstract

**Background:**

The clinical management of pancreatic cancer is severely hampered by the absence of effective screening tools.

**Methods:**

Sixty-seven biomarkers were evaluated in prediagnostic sera obtained from cases of pancreatic cancer enrolled in the Prostate, Lung, Colorectal, and Ovarian Cancer Screening Trial (PLCO).

**Results:**

The panel of CA 19-9, OPN, and OPG, identified in a prior retrospective study, was not effective. CA 19-9, CEA, NSE, bHCG, CEACAM1 and PRL were significantly altered in sera obtained from cases greater than 1 year prior to diagnosis. Levels of CA 19-9, CA 125, CEA, PRL, and IL-8 were negatively associated with time to diagnosis. A training/validation study using alternate halves of the PLCO set failed to identify a biomarker panel with significantly improved performance over CA 19-9 alone. When the entire PLCO set was used for training at a specificity (SP) of 95%, a panel of CA 19-9, CEA, and Cyfra 21-1 provided significantly elevated sensitivity (SN) levels of 32.4% and 29.7% in samples collected <1 and >1 year prior to diagnosis, respectively, compared to SN levels of 25.7% and 17.2% for CA 19-9 alone.

**Conclusions:**

Most biomarkers identified in previously conducted case/control studies are ineffective in prediagnostic samples, however several biomarkers were identified as significantly altered up to 35 months prior to diagnosis. Two newly derived biomarker combinations offered advantage over CA 19-9 alone in terms of SN, particularly in samples collected >1 year prior to diagnosis. However, the efficacy of biomarker-based tools remains limited at present. Several biomarkers demonstrated significant velocity related to time to diagnosis, an observation which may offer considerable potential for enhancements in early detection.

## Introduction

Pancreatic cancer is the fourth leading cause of cancer death in the United States. In 2013, an estimated 43,924 people will be diagnosed with pancreatic cancer and 37,390 will perish from the disease [1]. The high lethality associated with pancreatic ductal adenocarcinomas (PDAC), which constitute 85–90% of pancreatic cancer diagnoses, can be largely attributed to the presence of advanced disease at the time of diagnosis. PDAC is characterized by clinical manifestations which present late in the natural history of the disease at a stage when metastasis is a common finding, leading to a median survival of 6 months and an overall 5-year survival of <5% [2]. Outcomes are significantly improved in the minority of patients who present with small, surgically-resectable cancers for which there is a realistic chance of cure and a 5-year survival rate of 20–30% [3]. Considerable effort is currently devoted to the discovery and development of blood-based biomarkers capable of detecting PDAC at early, preclinical stages in appropriately targeted demographic groups.

Population-based screening for pancreatic cancer among asymptomatic individuals remains impractical based on the rarity of the disease and the lack of diagnostic tests with adequate accuracy. An effective screening test in this setting would require not only a high sensitivity (SN) for pancreatic cancer but also a level of specificity (SP) exceeding 99% in order to maintain an acceptably low level of false positive results. Screening programs targeting high-risk individuals are likely to be effective at more attainable performance standards due to the enrichment of PDAC prevalence within these populations. The mucin-associated sialylated Lewis (a) antigen CA 19-9 is a biomarker of PDAC shown to be ineffective as a standalone screening test. CA 19-9 has demonstrated modest effectiveness when applied as a diagnostic tool in symptomatic individuals on an outpatient basis with a median SN of 79% (range 70–90%) and median SP of 82% (range 68–91%), however it is not useful in the mass screening of asymptomatic subjects [4]. The principal limitations of CA 19-9 include its frequent elevation associated with pancreatitis and obstructive jaundice, conditions which frequently co-occur with pancreatic cancer and a variety of benign conditions.

The use of multiplex biomarker combinations has provided some advancement in the search for effective diagnostic tests for PDAC. Recent findings have generated interest in two potential biomarkers, osteopontin (OPN) and TIMP-1, in the early detection of pancreatic cancer [5]–[7]. TIMP-1 was also included in a three-biomarker panel along with CA 19-9 and carcinoembryonic antigen (CEA), which provided a SN/SP of 76/90 for the classification of pancreatic cancer from benign pancreatic disease [8]. In the same study, a panel comprised of CA 19-9, ICAM-1 and osteoprotegerin (OPG) provided a SN/SP of 88/90 for the discrimination of pancreatic cancer from healthy controls. Most recently, the combination of OPN, TIMP-1 and CA 19-9 was found to be effective in the discrimination of patients with pancreatic cancer from a group of healthy controls and patients diagnosed with pancreatitis [9]. The major limitation associated with these findings is the use of serum samples obtained near or after the time of PDAC diagnosis. Several groups have attempted the identification of PDAC biomarkers in pre-diagnostic samples. In a study by Faca et al., a panel of seven proteins with or without the addition of CA 19-9, selected based on findings in a mouse model, was able to discriminate human pancreatic cancer cases from matched controls in a small group of pre-symptomatic and pre-diagnostic subject included in the CARET (Carotene and Retinol Efficacy Trial) cohort [10]. Others have utilized larger prospective cohorts to separately implicate C-peptide levels and the IGF-1/IGFBP-3 ratio as markers of pancreatic cancer risk [11], [12].

In the current study we investigated the efficacy of a large group of serum biomarkers, including several combinations shown previously to be effective in a retrospective case/control cohort, in pre-diagnostic samples collected from patients diagnosed with PDAC who were enrolled in the large Prostate, Lung, Colorectal, and Ovarian Cancer Screening Trial (PLCO).

## Materials and Methods

### Ethics statement

All subjects involved in this study were over the age of 18 and provided written informed consent. Since signed informed consent was a criterion for eligibility to participate in the PLCO trial, each Screening Center determined the preliminary eligibility of potential participants and obtained their consent before enrolling them in the study. The Coordinator for each Screening Center was formally responsible for ensuring that informed written consent was obtained from each study participant. In addition, the Institutional Review Board (IRB) at each Screening Center approved the informed consent form(s) and specimen collection procedures. PLCO Screening Centers included the following: University Of Colorado, Georgetown University, Pacific Health Research & Education Institute, Henry Ford Health System, University Of Minnesota, Washington University, University Of Pittsburgh, University Of Utah, Marshfield Clinic Research Foundation, University Of Alabama At Birmingham, UCLA Immunogenetics Center. The current study was approved by the University of Pittsburgh IRB.

### Selection of cases and controls

The PLCO Cancer Screening Trial is a randomized multicenter trial in the United States, previously described in detail [13], which was aimed at evaluating the impact of early detection procedures for prostate, lung, colorectal, and ovarian cancer on disease-specific mortality. The study recruitment and randomization began in November 1993 and was completed in July 2001. The cohort comprised 152,810 men and women aged 55 to 74 years old at baseline.

Pancreatic cancer cases present among cohort participants were identified by self-report in annual mail-in surveys, state cancer registries, death certificates, physician referrals, and reports from next of kin for deceased individuals. All medical and pathologic records related to pancreatic cancer diagnosis and supporting documentation were obtained and abstracted by trained medical record specialists for cancer confirmation. Incident cases of primary adenocarcinoma of the exocrine pancreas (International Classification of Disease for Oncology, 3^rd^ edition code C250–C259) were included in current study. There were one hundred thirty-five incident cases of pancreatic adenocarcinomas between 1994 and 2006 (follow-up to 11.7 years; median, 5.4 years) confirmed through medical review. Controls, alive at the time when the index case was diagnosed, were randomly selected from all PLCO participants. Controls were matched to cases at a ratio of 4∶1 (controls:cases) based on the distribution of age (±5 years), race, sex, and calendar date of blood draw in 2-month blocks within the case cohort.

### Study design

Serum samples were provided by the PLCO administrators to UPCI in a blinded fashion for biomarker analysis (Table 1). According to the PLCO requirements, the analysis was performed in 5 steps as follows.

**Table 1:** Demographic and Clinical Characteristics of PLCO-selected Study Population.

	Training Set		Validation Set		Complete Set	
	Control	Case	Control	Case	Control	Case
n (%)	215 (100)	56 (100)	325 (100)	79 (100)	540 (100)	135 (100)
Gender						
Female	88 (41)	21 (38)	136 (42)	35 (44)	224 (42)	56 (42)
Male	127 (59)	35 (62)	189 (58)	44 (56)	316 (58)	79 (58)
Age						
≤59	31 (14)	15 (27)	67 (21)	12 (15)	98 (18)	27 (20)
60–64	68 (32)	12 (21)	95 (29)	23 (29)	163 (30)	35 (26)
65–67	69 (32)	20 (36)	94 (29)	28 (35)	163 (30)	48 (36)
≥70	47 (22)	9 (16)	69 (21)	12 (20)	116 (22)	25 (18)
BMI						
<30	165 (77)	39 (70)	239 (74)	59 (75)	404 (75)	98 (73)
30–40	44 (20)	16 (28)	82 (25)	16 (20)	126 (23)	32 (24)
>40	4 (2)	1 (2)	3 (1)	4 (5)	7 (1)	5 (4)
Unknown	2 (1)	0	1 (0.5)	0	3 (0.5)	0
Race						
White, non-hispanic	192 (89)	53 (95)	290 (89)	69 (87)	482 (89)	122 (90)
Black, non-hispanic	8 (4)	2 (4)	20 (6)	7 (9)	28 (5)	9 (7)
Hispanic	3 (1)	0	7 (2)	1 (1)	10 (2)	1 (1)
Asian	10 (5)	0	5 (2)	1 (1)	15 (3)	1 (1)
Pacific Islander	1 (0.5)	1 (2)	3 (1)	0	4 (1)	1 (1)
American Indian	0	0	0	1 (1)	0	1 (1)
Unknown	1 (0.5)	0	0	0	1 (0.1)	0
Smoking Status						
Never	96 (45)	23 (41)	145 (45)	28 (35)	241 (45)	51 (38)
Current	18 (8)	9 (16)	35 (11)	18 (23)	53 (10)	27 (20)
Former	101 (47)	24 (43)	145 (45)	33 (42)	246 (45)	57 (42)
Diagnosed with Diabetes						
Yes	21 (10)	7 (13)	23 (7)	12 (15)	44 (8)	19 (14)
No	194 (90)	49 (87)	301 (93)	67 (85)	495 (92)	116 (86)
Months to PDAC diagnosis						
<12 months		29 (52)		41 (52)		70 (52)
12+ months		27 (48)		38 (48)		65 (48)
Overall survival post-PDAC diagnosis						
<6 months		24 (43)		41 (52)		65 (48)
6–24 months		23 (41)		27 (34)		50 (37)
>24 months		9 (16)		11 (14)		20 (15)
Cancer Subtype						
Neoplasm		1 (2)		1 (1)		2 (2)
Carcinoma NOS		3 (5)		7 (9)		10 (7)
Adenocarcinoma NOS		43 (77)		61 (77)		104 (77)
Mucinous adenocarcinoma		2 (4)		2 (3)		4 (3)
Mucin-producing adenocarcinoma		1 (2)		1 (1)		2 (2)
Infiltrating duct carcinoma		6 (11)		5 (6)		11 (8)
Acinar cell carcinoma		0 (0)		1 (1)		1 (1)

#### Step 1. Initial training on a retrospective case/control set

The entire case/control set reported in [8] was used for training to identify optimal biomarker combinations, establish classification rules, and calculate scoring functions using the MMC algorithm (described below). This set consisted of 343 patients diagnosed with PDAC (163 men, 180 women, median age 68, age range 29–92) and 227 healthy controls (88 men, 139 women, median age 56, age range 18–87). The stage distribution for cases was 2.3% stage 1, 20% stage 2, 10% stage 3, 25% stage 4, and 39% unknown.

#### Step 2. Validation of an initial algorithm in the first, blinded one-half of the PLCO set

The first, blinded one-half of the PLCO set was analyzed for biomarkers included in the optimal combinations identified in Step 1. Two scoring functions determined in Step 1 were applied to this half of the PLCO set in order to assign diagnoses to each subject. These experimental diagnoses were then forwarded to the PLCO administrators for comparison to actual diagnoses and the diagnostic efficacy [SN, SP, area under the ROC curve (AUC)] of each biomarker combination was reported back to UPCI.

#### Step 3. Training on the first one-half of the PLCO set following unblinding

Once the results of the blinded PLCO training analysis were reported, the case/control status of those samples was unblinded in order to permit further biomarker analyses. This one-half of the set was evaluated for the entire panel of 67 biomarkers and used as a training set for development of improved algorithms.

#### Step 4. Validation of the results from Step 3 in the second, blinded one-half of the PLCO set

The improved algorithm was applied to the second blinded one-half of the PLCO set and scoring functions and diagnoses (cancer/healthy) were then forwarded to the PLCO. The experimental diagnoses were again compared to the actual diagnoses and the diagnostic efficacy (SN, SP, AUC) of each biomarker combination was reported back to UPCI.

#### Step 5. Training on the entire PLCO set

The entire PLCO set was unblinded, the full set of 67 candidate biomarkers were measured in all PLCO samples, and the entire dataset was utilized for development of a further optimized algorithm.

### Multiplexed Biomarker Analysis

A total of 67 multiplexed bead-based immunoassays targeting specific protein biomarkers were utilized over the course of the current study (Table 2). We previously reported the performance of several biomarker combinations in the discrimination of PDAC cases from healthy control subjects in a large retrospective case/control study [8]. In addition to biomarkers reported to be significantly altered in [8], a number of additional candidate biomarkers were analyzed, including AGRP, BDNF, CNTF, Cathepsin D, NCAM, MIC-1, MIP4, complement C4, clusterin, IGFBP3, periostin, and TTR. Assays targeting CEACAM-1, CEACAM-6, ALCAM, and HIF-1α were developed according to strict quality control standards by the UPCI Luminex Core Facility [14] and were performed as described previously [15]. Assays targeting TIMPs 1-4 and MMP-3 were obtained from R&D Systems (Minneapolis, MN) and all remaining assays were obtained from EMD Millipore (Billerica, MA). All commercially obtained assays were performed according to manufacturer instructions. The complete biomarker dataset has been deposited with the Early Detection Research Network (EDRN) of the National Cancer Institute (NCI) and can be accessed at https://edrn.jpl.nasa.gov/ecas/data/dataset/urn:edrn:Analysis_of_pancreatic_cancer_biomarkers_in_PLCO_set.

**Table 2:** Complete List of Evaluated Biomarkers.

Category	Biomarkers
Tumor Markers	ALCAM[20], AFP[21], CA 19-9[22], CA 72-4[23], CA125[24], CEA[25], CEACAM-1[26], CEACAM-6[27], CYFRA 21-1[28], HE4[29]
Acute Phase	Apo CIII[30], Apo E[31], Complement C4[31], CRP[32], transthyretin (TTR)[33]
Hormones	FSH[34], GH[34], bHCG[35], prolactin (PRL)[34]
Growth Factors	EGFR[34], ErbB2[34], FGF2[36], HGF[37], IGFBP-3[38], VEGFR1#, VEGFR2#, VEGFR3#, TGFα#,
Apoptosis	Fas[39], FasL[39], clusterin[40]
Cytokines/Inflammation	IL-8[41], MIF[34], MPO[34], IL-1R1#, IL-4R#, IL-6R#, TNFRI[34], TNFRII[34], YKL40#, MIC-1[59], MIP-4#, HIF-1α[42]
Metastasis	MMP-3[34], MMP-9[34], TIMP-1[43], TIMP-2#, TIMP-3#, TIMP-4#, tPAI1[34], NGAL[44]
Adhesion	ICAM-1[10], VCAM-1[34], NCAM[60], Periostin[45]
Gut Proteins	Cathepsin D[46], Insulin#, PTH[34]
Neural Factors	AGRP#, BDNF#, CNTF#, NSE#
Bone metabolic proteins	Osteocalcin (OC)[34], osteoprotegerin (OPG)[34], osteonectin (OSN)#, osteopontin (OPN)[34], TRAP5#

# - our unpublished observations

### Univariate and Multivariate Statistical Analysis

For the univariate analysis, biomarker measurements among the case and control groups were evaluated by the Mann-Whitney non-parametric U test. An initial minimum level of significance of p≤0.05 was utilized. The false discovery rate (FDR) was controlled at 5% according to the method of Benjamini and Hochberg[16]. Briefly, the individual p-values for each biomarker comparison were ranked from most to least significant. The ranked, unadjusted p-values were then compared to the statistic i*q/m, where i is the p-value rank, q is the FDR (0.05), and m is the total number of biomarker comparisons tested. Individual biomarker mean, SN, and AUC values were determined using Graphpad PRISM software (La Jolla, CA).

A Metropolis algorithm with Monte Carlo optimization (MMC) was used for the multivariate analysis of the biomarker results as described previously [17]. Briefly, all biomarker combinations of a predetermined size are examined. A scoring function (SF) is calculated for each biomarker panel as a linear combination of logarithms of biomarker concentrations multiplied by a coefficient for each biomarker assigned by Monte Carlo optimization. The resulting set of SFs for each biomarker combination is then evaluated for classification efficiency using 500× cross-validation. In order to avoid overfitting bias, our analysis was limited to panels consisting of 2, 3, or 4 biomarkers. In the Training analyses (Steps 3 and 5), panels were evaluated based on SN at predetermined SP levels of 95% and the statistical significance of differences in SN was assessed using McNemar's test for correlated proportions as described in [18]. A value for x^2^ of 3.841 offering a 5% significance level was used as a cutoff point. In the Training and Validation analyses (Steps 2–5), differences in AUC were assessed for significance as described by Hanley and McNeil [19]. Here, a Z ratio of ±2 was used as a cutoff point for statistical significance.

### Reproducibility of biomarker measurements and MMC scoring

The reproducibility of the biomarker measurements and the MMC algorithm was assessed through the inclusion of 17 duplicate samples embedded within the blinded PLCO set (7 cases/10 controls). Coefficients of variation (CV) were calculated for each duplicate set for each biomarker tested. Diagnoses determined by the MMC algorithm were compared within each duplicate set for consistency.

### Biomarker correlations with CA 19-9 levels and associations with time to diagnosis

Correlations between each of the biomarkers included in the current study and CA 19-9 were evaluated in the cases using the Pearson test of correlation with a minimum level of significance of p≤0.05. To assess the association between biomarker concentrations and time to diagnosis, biomarker levels were plotted against time to diagnosis measured in days for the complete set of cases from the PLCO set. Curves were evaluated by linear regression in order to identify those with non-zero slope values with a minimum level of significance of p≤0.05.

## Results

### Reproducibility

Coefficients of variation (CV) reflecting reproducibility in the measurement of 67 biomarkers, varied from 0% to 18.8% with average CV for each biomarker varying from 1.0% to 7.8% (Table 3 for nine representative biomarkers and data not shown). Diagnoses assigned by the MMC algorithm were consistent among each pair of duplicate samples.

**Table 3:** Reproducibility of measurements of representative biomarkers in duplicate PLCO samples.

	CA 19-9	CA 72-4	tPAI1	GH	BNDF	OPG	CEA	FSH	OC
CV range (%)	0.2–6.3	0.3–16.7	0.1–7.9	0.1–8.7	0.0–3.7	0.2–5.2	0.2–18.8	0.5–9.0	0.1–4.7
Mean CV (%)	1.9	5.9	2.3	2.6	1.2	1.7	5.6	2.6	2.0

### Multimarker panel discovery, training and validation

#### Step 1

In the retrospective case/control set, the combination of CA 19-9/OPG offered the highest performance of all two biomarker panels with a SN of 74.8% at 95% SP and an AUC value of 0.925, while the combination of CA 19-9, OPG, OPN offered the highest classification power of all 3-biomarker panels with SN = 82.4% at a SP = 95% and AUC = 0.954 (Table 4). None of the possible 4-biomarker panels offered a significant advantage over the CA19-9/OPG/OPN combination (data not shown). Therefore, the CA 19-9/OPG and CA 19-9/OPG/OPN panels were selected for evaluation in the prospective PLCO cohort.

**Table 4:** Performance of Multimarker Combinations in PLCO Training and Validation Sets.

**Step 1. Training Case/Control Set** *			
	SN/SP/AUC		
CA 19-9/OPG	74.8/95.0/.925		
CA 19-9/OPG/OPN	82.4/95.0/.935		
**Step 2. Validation on blinded first-half of PLCO set**			
	Complete Set	MTD 1–12	MTD 12–35
	SN/SP/AUC	SN/SP/AUC	SN/SP/AUC
CA 19-9	23.2/90.2/.652	20.7/90.2/0.669	25.9/90.2/.633
CA 19-9/OPG	32.1/87.8/.570	31.0/87.8/.614	33.3/87.8/.523
CA 19-9/OPG/OPN	30.4/84.7/.547	37.9/84.7/.596	22.2/84.7/.494
**Step 3. Training on unblinded first-half of PLCO set**			
	Complete Set	MTD 1–12	MTD 12–35
			
CA 19-9	17.9/95.0/.652	17.2/95.0/.669	18.5/95.0/.633
CA 19-9/CEA	30.4#/95.0/.665	42.3#/95.0/.749	20.0/95.0/.555
**Step 4. Validation in blinded second-half of PLCO set**			
	Complete Set	MTD 1–12	MTD 12–35
	SN/SP/AUC	SN/SP/AUC	SN/SP/AUC
CA 19-9	25.3/93.2/.656	38.1/93.2/.695	10.8/93.2/.616
CA 19-9/CEA	31.7/94.4/.668	40.5/94.4/.710	21.8/94.4/.620
**Step 5. Training on the entire unblinded PLCO set**			
	Complete Set	MTD 1–12	MTD 12–35
	SN/SP/AUC	SN/SP/AUC	SN/SP/AUC
CA 19-9	21.8/95.0/.656	25.7/95.0/.680	17.2/95.0/.626
CA 19-9/CEA	28.1^/^95.0/.616	26.7^/^95.0/.666	28.1#/95.0/.657
CA 19-9/CEA/Cyfra 21-1	30.4#/95.0/.678	32.4#/95.0/.692	29.7#/95.0/.663

*Case/Control set described in [8].

# Statistical significance of differences in SN in comparison with CA 19-9 alone, method descrived in [18].

SN/SP/AUC – sensitivity/specificity/area under ROC curve.

MTD 1–12 – months to diagnosis 1–12, samples collected <12 months prior to diagnosis.

MTD 12–35 – months to diagnosis 12–35, samples collected 12 to 35 months prior to diagnosis.

#### Step 2

Overall, the performance of selected panels was markedly diminished in the pre-diagnostic PLCO samples in comparison to the case/control samples obtained at the time of diagnosis (Table 4). In the complete set as well as in subsets including cases diagnosed within 12 months of sample collection [months to diagnosis (MTD) 1–12 group] and those diagnosed 12–35 months after collection (MTD 12–35 group), both the CA 19-9/OPG and CA 19-9/OPG/OPN biomarker combinations provided statistically similar AUC values, which in turn did not differ significantly from that of CA 19-9 alone.

#### Step 3

Next, the MMC algorithm was applied to the unblinded one-half of the PLCO set in which a larger pool of 67 candidate biomarkers was measured. All SN values were determined at 95% SP in order to approximate the requirements for high SP in PDAC screening. CA 19-9 alone offered 17.9% SN at 95% SP in the entire set with 17.2% SN in the MTD 1-12 group and 18.5% SN in the MTD 12–35 group. The combination of CA 19-9/CEA performed better than CA 19-9 alone and provided a SN of 30.4% at 95% SP for the entire set and SNs of 42.3% and 20% for the MTD 1-12 and MTD 12–35 groups, respectively. Differences in SN of the CA 19-9/CEA combination vs. CA 19-9 alone reached statistically significant levels when assessed in the entire set and in MTD 1–12 group (Table 4).

#### Step 4

Two sets of diagnoses for the second, blinded one-half of the PLCO set were forwarded to the PLCO administrators using the following classifiers: CA 19-9 alone and CA 19-9/CEA.

The combination of CA 19-9/CEA provided somewhat elevated levels of SN, SP and AUC over CA 19-9 alone (Table 4), however the observed differences in AUC did not reach statistical significance.

#### Step 5

**Figure 1:**
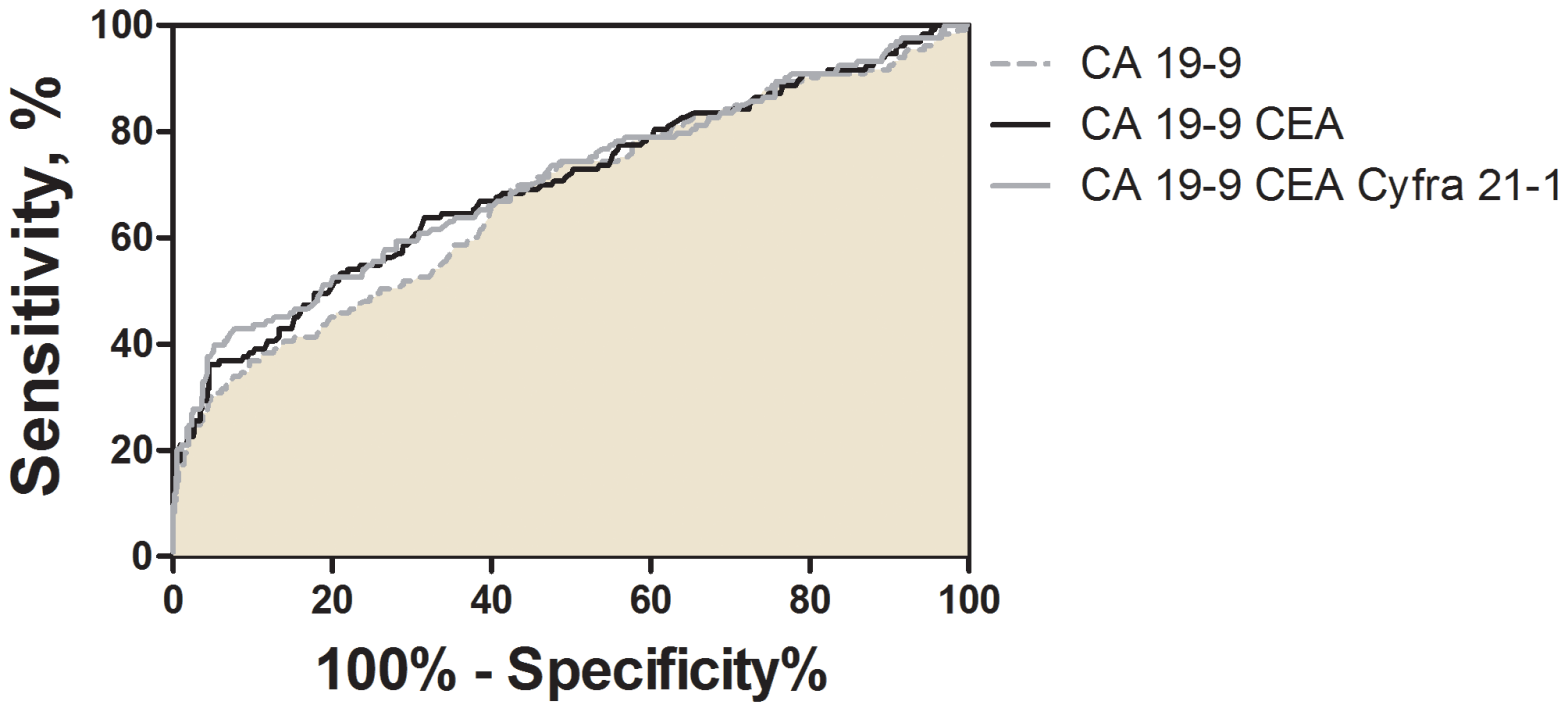
Biomarker panel performance in the complete PLCO cohort. A Metropolis algorithm with Monte-Carlo simulation was utilized to identify the top performing biomarker combinations in the discrimination of PDAC cases from matched controls within the PLCO cancer screening trial. ROC curves reflecting the performance of CA 19-9, the top two biomarker panel (CA 19-9/CEA), and the top three biomarker panel (CA 19-9/CEA/Cyfra 21-1) are shown. AUCs for the three models did not differ significantly according to the method of Hanley and McNeil [19].

Finally, the entire PLCO set was unblinded for training using the complete panel of 67 biomarkers. The MMC algorithm was used to evaluate all possible combinations of 2, 3 and 4 biomarkers in the complete PLCO cohort at a preset SP of 95%. CA 19-9 alone was 21.8% sensitive in the entire set, with 25.7% SN in the MTD 1–12 group and 17.2% SN in the MTD 12–35 group (Table 4). As expected, among all possible 2-biomarker panels, the CA 19-9/CEA combination had the highest diagnostic power with 28.1% SN in the entire set, 26.7% SN in the MTD 1–12 group, and 28.1% in the MTD 12–35 group. Of all evaluated 3-biomarker combinations, the combination of CA 19-9/CEA/Cyfra 21-1 offered some advantage with an overall SN of 30.4%, a SN of 32.4% in the MTD 1–12 group, and a SN of 29.7% in the MTD 12–35 group, all at 95% SP. The CA 19-9/CEA combination demonstrated higher levels of SN in comparison to CA 19-9 alone in the complete set and the MTD 1–12 set, however neither of these differences were statistically significant. The CA 19-9/CEA panel provided a significantly improved SN over CA 19-9 alone in the MTD 13–35 set. The combination of CA 19-9/CEA/Cyfra 21-1 provided significantly improved SN levels in all three case sets. ROC curves demonstrating the performance of each of the top biomarker panels and CA 19-9 alone are presented in Figure 1.

#### Individual biomarker performance

**Figure 2:**
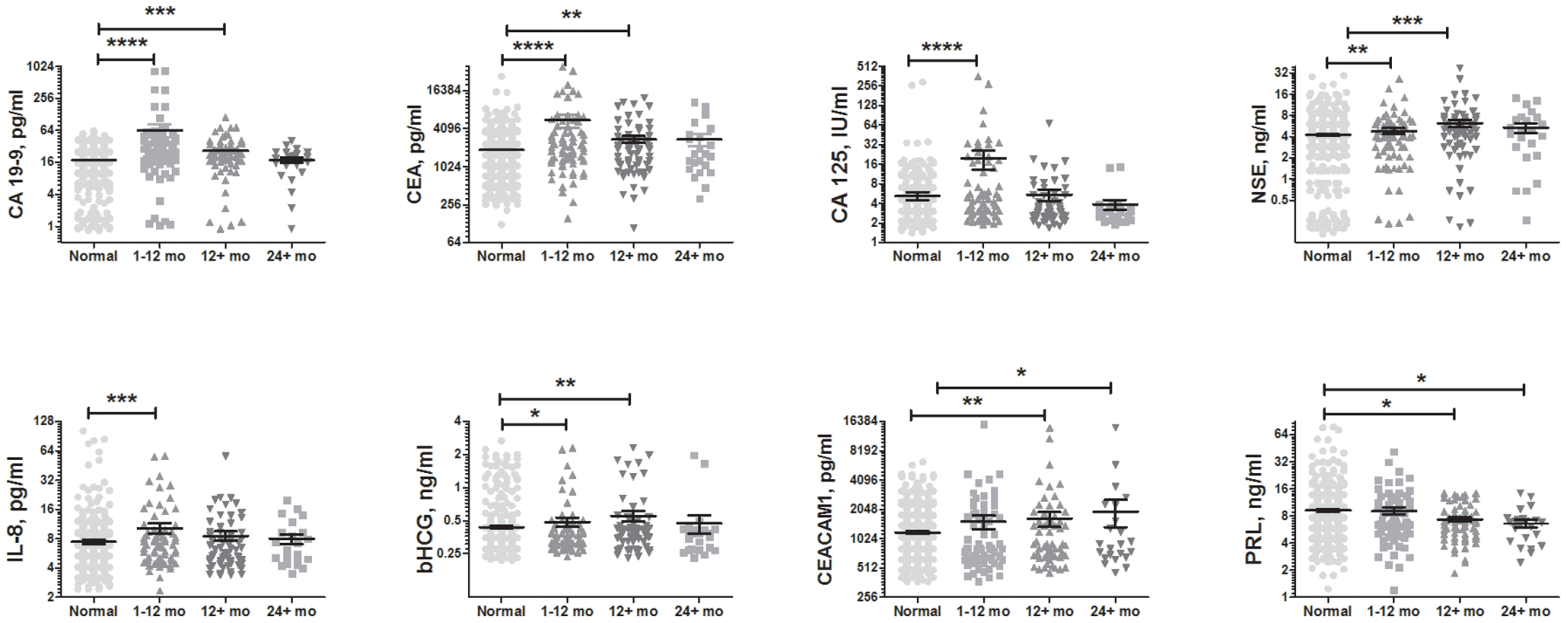
Prediagnostic distributions of serum biomarker levels. Levels of 67 biomarkers were evaluated in sera obtained from 135 subjects enrolled in the PLCO cancer screening trial who were subsequently diagnosed with pancreatic cancer and 540 matched controls. Circulating levels of biomarkers demonstrating significant differences between cases and healthy controls are presented. Level of significance: * - p<0.03, ** - p<0.01, *** - p<0.001, **** - p<0.0001.

Upon completion of the current study, a total of 67 biomarkers were evaluated in the full unblinded PLCO set. Among these, eight biomarkers were found to differ significantly among the case and control groups according to the MWU test: CA 19-9, CEA, CA 125, NSE, CEACAM1, IL-8, PRL, and bHCG (Figure 2, Table 5). After controlling for a false discovery rate of 5%, the level of significance was set at p<0.03. Each significantly altered biomarker was observed at higher levels in cases than in controls, with the exception of PRL, which was observed at lower levels in the cases. CA 19-9, CEA, NSE, and bHCG demonstrated differences in both the MTD 1-12 and MTD 12-25 subsets, whereas differences in CA 125 and IL-8 reached statistical significance in only the MTD 1–12 subset. Differences in CEACAM1 and PRL were significant in the 12–35 MTD and 24–35 MTD groups but not in the 1–12 MTD groups.

**Table 5:** Individual Performance of Significantly Altered Serum Biomarkers.

	PDAC (pg/ml)			Healthy (pg/ml)			PDAC vs. Healthy		
	Low	High	Mean	Low	High	Mean	Cut-point	SN (%)	AUC
CA 19-9	0.173	4600	55.7	0.155	6220	25.0	4.40	21.8	0.656
CEA	199	47700	3160	124	2090000	8420	8980	6.67	0.525
CA 125	1.52	1060	44.2	1.06	2790	28.2	86.1	6.67	0.574
NSE	209	37400	5480	165	29500	4250	10600	8.89	0.577
IL-8	2.34	56.5	9.47	2.43	102	7.47	14.7	15.0	0.596
bHCG	0.230	2.30	0.518	0.218	2.65	0.435	1.19	9.16	0.579
CEACAM1	372	15400	1580	372	6260	1180	2900	9.70	0.535
Prolactin	4060	451000	39500	5490	1440000	46900	12700	16.1	0.570

Cut-point – minimum (maximum for prolactin) value (pg/ml) for diagnosis as case at 95% specificity.

SN – sensitivity at 95% specificity.

AUC – area under ROC curve.

#### Biomarker associations with time to diagnosis and correlations with CA 19-9 levels

**Figure 3:**
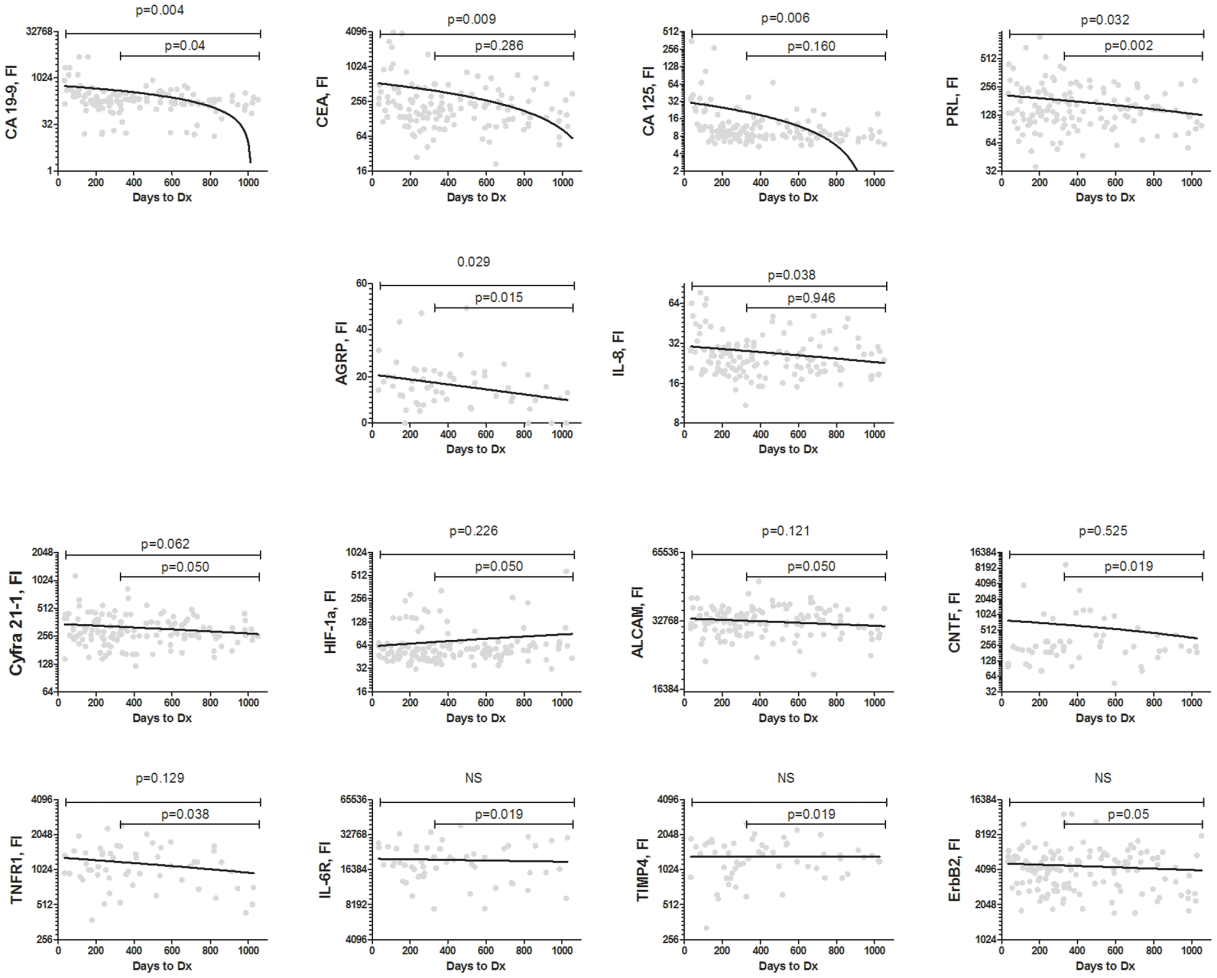
Biomarker levels in relation to time to diagnosis. Biomarker levels were plotted against the elapsed time interval between blood draw and cancer diagnosis and plots were evaluated by linear regression. Biomarkers demonstrating slopes differing significantly from zero are presented.

Levels of CA 19-9, CA 125, CEA, PRL, AGRP, and IL-8 demonstrated negative associations with time to diagnosis with linear regression slopes differing significantly from zero (Figure 3). Importantly, CA 19-9, PRL, and AGRP showed slopes significantly differing from zero in samples collected more than 12 months before diagnosis. Additionally, several biomarkers (Cyfra 21-1, TNFR1, ErbB2, CNTF, IL-6R, HIF-1a, TIMP-4 and ALCAM) demonstrated regression slopes which were significant only when analyzed in samples collected more than 12 months before diagnosis, but not in the overall set (Figure 3). Only CA 125 (r^2^ = 0.5361, *p*<.0001) and CEA (r^2^ = 0.6947, *p*<.0001) were observed to be significantly correlated with CA 19-9.

## Discussion

We report here a systematic analysis of 67 serum protein biomarkers in pre-diagnostic samples collected from patients diagnosed with PDAC in the course of the PLCO study. Published reports and our previous case/control analysis utilizing sera drawn at the time of pancreatic cancer diagnosis yielded a broad and diverse spectrum of biomarker alterations [20]–[46]. From these results, we concluded that local and systemic responses to tumor progression in advancing and/or symptomatic disease resulted in an extensive milieu of factors detectable in the sera of pancreatic cancer patients. The results of our current study reflect the challenges associated with the detection of early asymptomatic disease in that only a subtle array of 8 biomarker alterations was observed. Our analysis of biomarker level trends across the prediagnostic course of PDAC indicate that CEACAM1 and PRL are the earliest to be detected at significantly altered levels at time points up to 35 months prior to diagnosis. Subsequently, changes in CA 19-9, CEA, NSE, and bHCG are observed that are detectable up to 24 months before diagnosis. Finally, levels of CA 125 and IL-8 are detectably elevated up to 12 months before diagnosis. Importantly, these results demonstrate the limitations of using CA 19-9 as a biomarker for very early or pre-neoplastic disease.

Our results suggest that several circulating PDAC biomarkers which have been identified in case/control studies, including MIC-1, TIMP-1, ICAM1, HE4, OPG, MUC1, MMP9, SAA, and others [10], [34], [47], may not be useful for prediagnostic risk assessment. However, as a number of biomarkers differentially expressed in pre-diagnostic samples in the current study (CA 19-9, CEA, CA 125, CEACAM1, IL-8, PRL, and bHCG), were initially reported for case/control studies [23], [26], [34], [48], the concept that case/control setting are appropriate for initial identification of biomarker candidates may prove valid in highly selective instances. Therefore, ongoing efforts should be aimed at the validation of circulating levels of additional biomarkers shown to be differentially expressed in pancreatic tumors and preneoplastic lesions, such as those summarized in [49], in preclinical serum/plasma samples.

While the high performing panels identified in our previous case/control analysis failed to perform adequately in the current study, the identification of potential alternative panels should provide a sound basis for further development of screening tools. In the training/validation phase of the study, the combination of CA 19-9/CEA performed best, although a statistically significant advantage over CA 19-9 alone was not achieved in the validation set. The inclusion of CEA was somewhat surprising given the observation that levels of CEA were significantly correlated with those of CA 19-9, thus limiting its potential for diagnostic complementation. However, CEA has been previously noted for its relatively high SP but low SN for PDAC [48], [50], a trend opposite that of CA 19-9 and it may be that enhancements in SP led to the efficacious performance of this combination. In the unblinded analysis of the entire PLCO set, the combination of CA 19-9, CEA, and Cyfra 21-1 provided the highest level of performance with over 30% of cases correctly identified at 95% SP.

According to a recently described computational model of the clonal evolution of PDAC development, 6.8 years elapse between the development of a malignant clone and metastasis implying that the window for early detection and intervention is wider than initially believed [51], [52]. The temporal expression pattern of PDAC biomarkers described here, with changes occurring up to 35 months before diagnosis, indicate the presence of a systemic PDAC signature at pre-metastatic stages. Based on the computational model of PDAC progression, the use of the biomarker panels identified here as screening tools would likely identify some, but not all cases of PDAC prior to the development of metastasis. Although the clinical impact of such a screening strategy remains to be assessed, these findings do suggest that a higher rate of detection of resectable disease, associated with better outcomes, may be attainable in certain high risk groups. Groups at high risk for pancreatic cancer include families identified with Peutz-Jeughers syndrome (relative risk of 132), hereditary pancreatitis (relative risk of 50 to 67), familial atypical multiple mole melanoma (relative risk of 13 to 39), hereditary nonpolyposis colorectal cancer (relative risk of 8.6), familial adenomatous polyposis (relative risk of 4.5), breast and ovarian cancer syndrome (relative risk of 2 to 9), and individuals with multiple first-degree relatives diagnosed with PDAC [53]. Screening among these groups remains feasible with several groups reporting recent efficacy utilizing imaging and CA 19-9/CEA based strategies (reviewed in [54]).

Our analysis provides evidence that several biomarkers demonstrate significant velocity related to time to diagnosis of PDAC. A statistical model based on the velocity of serial CA 125 serum measurements in ovarian cancer patients, termed the Risk of Ovarian Cancer Algorithm (ROCA), has demonstrated efficacy in the early detection of ovarian cancer by offering a significant enhancement in SN in comparison to single measurements of CA 125 [55]. A simulated study also indicated that ROCA may double the SN of CA 125 for early stages of ovarian cancer (S. Skates, personal communication). Two of the biomarkers included in the top performing panel, CA 19-9 and CEA, demonstrated significant velocity related to time to diagnosis, suggesting that serial measurement of these biomarkers may lead to similar enhancements in panel performance. A study regarding the resectability of presymptomatic pancreatic cancer in diabetic patients indicated that a short window of several years exists when tumors of the pancreas can be visualized by CT and resected [56]. The striking finding in this study is the rapid progression from a normal pancreas to an unresectable tumor. These findings, combined with the molecular model of PDAC progression [52], suggest that specimens collected 1 to 4 years prior to diagnosis would likely lead to the successful identification of candidates for surgical resection. The identification of several biomarkers which selectively demonstrate velocity in sera collected over 1 year prior to diagnosis suggest the presence of biomarker signatures which may be specific for resectable disease. However, these conclusions regarding the efficacy of biomarker velocity in PDAC detection are preliminary and will require further study.

In the study by Palaez-Luna et al. [56] cited above, new onset diabetes was found to be associated with resectable PDAC, however several other studies have reported contradictory findings [57], [58]. In the current study, diabetes prevalence (± SEM) differed between cases (14.1±1.10%) and controls (8.0±3.00%) with p = 0.035 (unpaired t test). Also, diabetes rates were significantly different between cases demonstrating an overall survival of less than 6 months (16.7±4.62%), and those with an overall survival of greater than 12 months (0.0±0.00%) with p = 0.005. Although our analysis does not differentiate between new onset and chronic diabetes, these anecdotal findings do support the association between diabetes and PDAC and a particular association with unresectable disease.

Limitations to the current study include the limited extent of diagnostic interval present in our case group. Samples collected beyond 3 years prior to PDAC diagnosis were not available or admissible according to our selection criteria. Such cases would indeed be useful given the extended prediagnostic window suggested by the recent computational modeling of PDAC cited above. Other cohort studies with prospectively collected biospecimens with long-term follow-up could offer the opportunity to address this limitation. The current findings were also limited in that the highest performing biomarker panel was identified through the use of the entire subject cohort as one large training set. Thus, the performance of that panel will need to be further assessed through independent validation studies.

In conclusion, an analysis of a large array of serum biomarkers in prediagnostic PDAC patients enrolled in the PLCO Cancer Screening trial indicates that many biomarkers identified previously in retrospective case-control studies do not provide efficacy in this setting. Our findings do identify several alternative biomarkers observed to be altered in prediagnostic samples and several biomarker combinations capable of discriminating cases from controls as far as 2-3 years prior to diagnosis. Further efforts are necessary to expand the analysis of circulating biomarkers shown to be differentially expressed in pancreatic tumors and preneoplastic lesions in preclinical serum/plasma samples. Finally, these results suggest that the performance of biomarker-based screening tools is considerably limited at this stage of development and implementation should be limited to well-characterized high risk groups.
