# Correction: Prediagnostic Serum Biomarkers as Early Detection Tools for Pancreatic Cancer in a Large Prospective Cohort Study

**DOI:** 10.1371/journal.pone.0117876

**Published:** 2015-02-06

**Authors:** The PLOS ONE Staff

Ying Huang is not included in the author byline. She should be listed as the eighth author and affiliated with Fred Hutchinson Cancer Research Center, University of Washington, Seattle, WA, United States. The contributions of this author are as follows: Analyzed the data.

## References

[1] Nolen BM, Brand RE, Prosser D, Velikokhatnaya L, Allen PJ, et al (2014) Prediagnostic Serum Biomarkers as Early Detection Tools for Pancreatic Cancer in a Large Prospective Cohort Study. PLoS ONE 9(4): e94928 doi:10.1371/journal.pone.0094928 24747429 10.1371/journal.pone.0094928 PMC3991628

